# Pilot Study on AI Image Analysis for Lower-Limb Reconstruction—Assessing ChatGPT-4’s Recommendations in Comparison to Board-Certified Plastic Surgeons and Resident Physicians

**DOI:** 10.3390/life15010066

**Published:** 2025-01-08

**Authors:** Silke Graul, Michael A. Pais, Rafael Loucas, Tobias Rohrbach, Elias Volkmer, Sebastian Leitsch, Thomas Holzbach

**Affiliations:** 1 Department of Hand and Plastic Surgery, Thurgau Hospital Group, 8501 Frauenfeld, Switzerland; 2 Department for BioMedical Research, University of Bern, 3012 Bern, Switzerland; 3 Department of Plastic, Hand and Reconstructive Surgery, University Hospital Regensburg, 93053 Regensburg, Germany; 4 Australian Centre of Health Engagement, Evidence and Values (ACHEEV), University of Wollongong, Wollongong 2500, Australia; 5 Department of Hand Surgery, Helios Klinikum Munich West, 81241 Munich, Germany

**Keywords:** ChatGPT, ChatGPT-4, image analysis, artificial intelligence, AI, survey, lower-limb reconstruction, reconstructive surgery, plastic surgery

## Abstract

AI, especially ChatGPT, is impacting healthcare through applications in research, patient communication, and training. To our knowledge, this is the first study to examine ChatGPT-4’s ability to analyze images of lower leg defects and assesses its understanding of complex case reports in comparison to the performance of board-certified surgeons and residents. We conducted a cross-sectional survey in Switzerland, Germany, and Austria, where 52 participants reviewed images depicting lower leg defects within fictitious patient profiles and selected the optimal reconstruction techniques. The questionnaire included cases with varied difficulty, and answer options did not always include the most obvious choices. Findings highlight that ChatGPT-4 successfully evaluated various reconstruction methods but struggled to determine the optimal solution based on the available information in visual and written forms. A chi-squared test of independence was performed to investigate the overall association between answer options (A, B, C, and D) and rater group (board-certified surgeons, ChatGPT-4, and resident). Inter-group rater associations showed significant overall test results (*p* < 0.001), with high agreement among board-certified surgeons. Our results suggest that board-certified plastic surgeons remain essential for patient-specific treatment planning, while AI can support decision-making. This reaffirms the role of AI as a supportive tool, rather than a replacement, in reconstructive surgery.

## 1. Introduction

In recent years, the integration of artificial intelligence (AI) into various fields has impacted traditional practices, with the healthcare sector being no exception [1]. Artificial intelligence (AI) has emerged as a transformative tool in medicine, leveraging advanced language models to enhance clinical workflows, patient education, and administrative efficiency [2]. These technologies offer the potential to improve human–computer interactions, providing scalable solutions to complex medical challenges while raising critical questions about their integration into healthcare practices [2].

Among the AI innovations, ChatGPT (Chat Generative Pre-Trained Transformer), developed by OpenAI (San Francisco, CA, USA), stands out as a prominent tool designed to enhance human–computer interactions through its conversational capabilities [2]. ChatGPT is built on OpenAI’s proprietary series of generative pre-trained transformer (GPT) models, specifically fine-tuned for conversational applications using a blend of supervised learning and reinforcement learning from human feedback [3]. The latest generation, ChatGPT-4, is a multimodal model capable of processing both text and images as input, which enhances its ability to engage in more natural interactions and provide detailed responses based on visual information [4]. In the field of plastic surgery, conducting a literature search, Sharma et al. 2023 revealed several applications for ChatGPT like contributing to patient care by generating literature, aiding communication, assisting with administrative tasks, and supporting education and training for medical professionals [5].

Despite its advancements, ChatGPT-4, like its predecessors, retains certain limitations, such as generating information not found in the training data or contradicting user prompts [6]. Additionally, ChatGPT-4 lacks transparency in its decision-making processes [7]. It is crucial for medical professionals and patients to verify AI-generated medical information against trusted sources, underscoring the importance of ongoing evaluation [8]. ChatGPT-4 has been evaluated in a range of surgical scenarios, including plastic surgery and hand surgery, where it has received high performance ratings from expert surgeons [9].

The increasing accessibility of AI tools necessitates the careful consideration of their potential applications in surgical specialties. Evidence-based research is essential to guide the effective implementation of this resource into practice. Our focus was to determine whether ChatGPT-4 could generate practical suggestions based on image analysis through a brief case report and be considered a supportive tool for decision-making in plastic surgery.

Therefore, this study aims to determine whether ChatGPT-4 can provide plausible recommendations for the reconstruction of lower leg defects by analyzing images and the processing relevant clinical information in patients with varying pre-existing medical conditions. To our knowledge, this is the first study to focus on the image analysis capabilities of ChatGPT-4 in cases of defect injuries. We compared the AI-generated recommendations with the responses from colleagues with different levels of experience, including board-certified surgeons and residents in training. The objective was to evaluate whether ChatGPT-4 can serve as a reliable support tool in the decision-making process within the reconstructive plastic field, or if trained and certified plastic surgeons remain irreplaceable in routine clinical practice. The reported differences between the recommendations of ChatGPT-4 and experienced surgeons emphasize the essential role of expert judgment in reconstructive surgery. This aligns with the current literature, which shows that in complex cases, surgeons rely on their nuanced understanding of patient conditions and disease progression—an expertise that current AI models, constrained by the limited contextual depth of their training data, are unable to replicate [8,9].

## 2. Materials and Methods

We conducted a cross-sectional questionnaire study targeting hospitals in Switzerland, Germany, and Austria. An online survey was distributed via email to 570 board-certified plastic surgeons and residents in plastic surgery departments across Germany, Austria, and Switzerland. All authorized trainers in plastic surgery and their departments were contacted. Additionally, secretariats were asked to forward the survey to their employed board-certified plastic surgeons and residents in plastic surgery.

All participants were asked to select the optimal reconstruction technique for lower leg defects based on defined options. The focus of the study was on image analysis. The images depicted lower leg defects in the context of fictitious patient profiles, which included various mechanisms of injury and patients of different ages, as well as diverse pre-existing comorbidities such as vascular occlusions and diabetes. The case reports were selected to represent varying levels of difficulty. The answer options did not always include the most obvious choices, aiming to evaluate participants’ depth of understanding and probe their clinical experience. The questionnaire was designed to be anonymous to encourage a higher response rate and more thoughtful answers, thereby ensuring unbiased data. Additionally, the survey inquired about the current range of subspecialties offered in hospitals across Switzerland, Germany, and Austria.

### 2.1. Baseline Demographic Data

The participant-related data collected included gender and training status, differentiating between board-certified surgeons and residents in training. Among the board-certified surgeons, we distinguished those with more than three years of practice post-certification from those with less than three years of experience. Residents were categorized based on their years of training following the completion of their surgical common trunk education. The surgical specialties of both board-certified surgeons and residents encompassed esthetic surgery, reconstructive surgery, microsurgery, and hand surgery.

### 2.2. Design of Questionnaire

**Figure 1:**
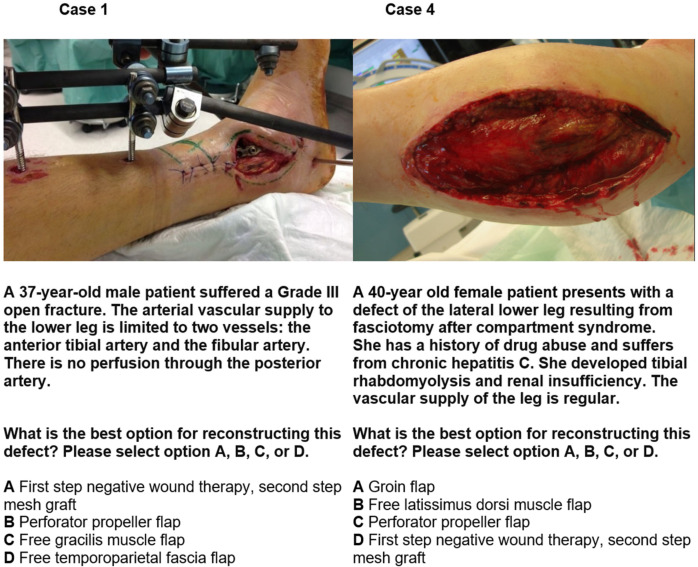
Example case report 1 and 4. In case 1, 95% of board-certified surgeons, 50% of residents, and ChatGPT-4 selected answer C. In case 4, 97% board-certified surgeons and 90% of residents selected answer D. ChatGPT-4 opted for answer C.

The questionnaire was entered into the UmfrageOnline (enuvo GmbH, Zürich, Switzerland) system. The cover letter was performed in three languages: German, Italian, and French. The questionnaire was made available in English. In the first part of the survey, the participants were asked to give their gender, status of training, work experience, and their plastic-surgical spectrum (questions 1, 2, and 3). The second part presented a series of four constructed case reports with various accident mechanisms and previous illnesses. The accompanying information on each case was deliberately kept brief (see, for example, Cases 1 and 4, Figure 1). All participants were asked to pick the best possible answer of optimal reconstruction of lower leg defect based on the image and given information. We compared the results with the answers from colleagues with different levels of experience and gave the same information to ChatGPT-4 (accessed on 14 April 2024, https://chatgpt.com/) to analyze the given information and image. We treated ChatGPT-4 as a single participant with one consistent opinion. It was configured with standard settings and no preloaded knowledge to minimize bias. The memory function was deactivated.

### 2.3. Case Reports/Questionnaire

We presented four fictitious case reports, each with four answer options labeled A to D. The images depicted lower leg and foot defects to demonstrate variations in severity levels. The case reports were kept concise, and the answer options did not always include the most obvious choices. We always provided information regarding vascular supply.

The research committee of Spital Thurgau HPC approved this study. Additional approval was deemed unnecessary as the study utilized anonymized patient images and fictitious case reports, thereby maintaining confidentiality and ethical standards. Our methodology adheres to the 1964 Declaration of Helsinki and its subsequent amendments, ensuring ethical integrity and the protection of patient privacy.

Figure 1 shows Cases 1 and 4 as an example, including the image, fictitious case report, question, and answer options.

### 2.4. Statistical Analysis

The participant’s information was anonymized. The different reconstruction possibilities were compared using a frequency distribution table for descriptive statistics of data as discussed above. Chi-squared tests of independence were used to investigate the overall association between answer options (A, B, C, and D) and rater group (board-certified surgeons, ChatGPT-4, and resident). The data included binary variables, indicated as a percentage of the total positive counts [pct]. Significant results in the overall tests were followed up with post hoc pairwise group comparisons with Bonferroni adjusted *p* values to control for the increased risk of Type I errors due to multiple comparisons. Statistical significance was considered for *p* values < 0.05. Statistical analyses were conducted using GraphPad Prism (v9.5.1, GraphPad Software, San Diego, CA, USA).

## 3. Results

### 3.1. Baseline Demographic Data and Questionnaire Results

Complete datasets were collected from 52 of the 570 board-certified plastic surgeons and residents in plastic surgery departments across Switzerland, Germany, and Austria, resulting in a response rate of 9%. Among the participants, 32 were male, 18 female, and one identified as diverse gender. Overall, 75% were specialists, while 25% were residents in plastic, reconstructive, and esthetic surgery. Almost all board-certified plastic surgeons engaged in reconstructive surgery (97%) and micro-surgery (82%), with 77% also performing esthetic procedures. Approximately two-thirds (62%) of the surgeons conducted hand surgery, and about one-third performed burn surgery. All residents performed reconstructive surgery, with 77% engaging in microsurgery, and 69% in esthetic, hand, and burn surgery. The majority of specialists (90%) reported having over three years of experience following board certification, while the average experience among residents was three years post-common trunk training (Table 1).

### 3.2. ChatGPT-4 Questionnaire Results

ChatGPT-4 was presented with the same questionnaire given to our board-certified surgeons and residents. It was tasked with selecting the most appropriate answer for the optimal reconstruction of lower leg defects, utilizing image analysis and processing relevant additional information to evaluate pre-existing medical conditions. Even when asked multiple times, the answer remained consistent.

### 3.3. Comparison of Questionnaire Results

#### 3.3.1. Case 1

In Case 1, ChatGPT-4 (100%), residents (50%) and board-certified surgeons (95%) selected answer C as the best option for reconstructing the defect. Residents showed the most variation, with 10% choosing A and 40% choosing B. Only 5% of board-certified surgeons selected B (Table 2).

#### 3.3.2. Case 2

In Case 2, ChatGPT-4 (100%) selected answer B, in contrast to no residents or board-certified surgeons choosing this option. Residents (60%) and board-certified surgeons (61%) selected answer C as the best option for reconstructing the defect. Forty percent of the residents answered with D, while among board-certified surgeons, 21% chose D, and 18% selected A (Table 3).

#### 3.3.3. Case 3

In Case 3, ChatGPT-4 (100%), a minority of residents (10%), and board-certified surgeons (11%) selected answer B as the best option for reconstructing the defect. The majority of board-certified surgeons (66%) chose A as the best option, while 18% chose C and 5% chose D. Among residents, 40% selected A and 40% selected C, while 10% chose B, and 10% chose D (Table 4).

#### 3.3.4. Case 4

In Case 4, ChatGPT-4 (100%) chose answer C, while only 10% of residents selected this option. The majority of residents (90%) and board-certified surgeons (97%) selected answer D as the best option for reconstructing the defect. Only 3% of board-certified surgeons selected answer A (Table 5).

#### 3.3.5. Inter-Group Rater Associations

**Figure 2:**
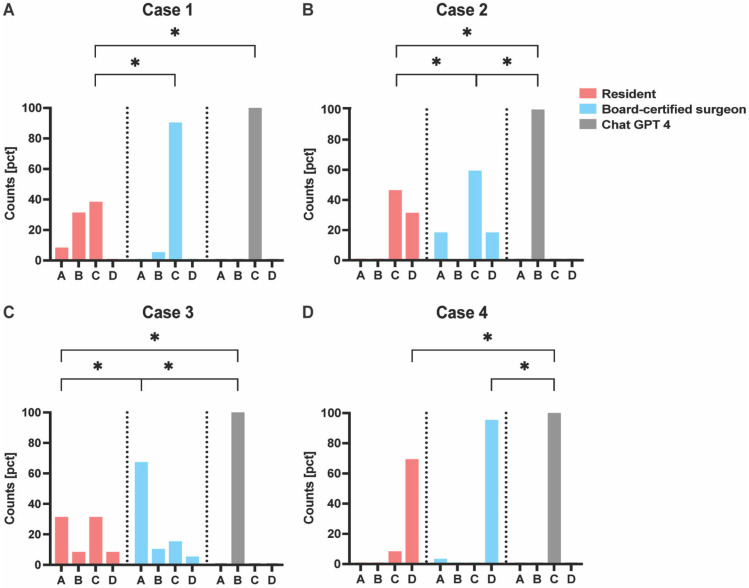
Inter-group rater associations with chi-squared test. A, B, C, D = answers, Binary variables. * = significant difference, pct = positive counts.

A chi-squared test of independence was performed to investigate the overall association between answer options (A, B, C, and D) and rater group (board-certified surgeons, ChatGPT-4, and residents). The data are binary variables and indicated as a percentage of the total positive counts [pct] (Figure 2). Inter-group rater associations show high agreement among board-certified surgeons.

The following was analyzed in detail for each case:

A. Case 1 was the only case where ChatGPT-4, residents, and board-certified surgeons selected the same answer. Fewer residents selected answer C, which was followed by significant differences between board-certified surgeons and residents (*p* < 0.003), as well as between residents and ChatGPT-4 (*p* < 0.003), respectively. Overall tests showed significant results (*p* < 0.001).

B. In Case 2, the most common answer was C, selected by both board-certified surgeons and residents, which was significantly different from ChatGPT-4’s response. Although there was also a difference between the group of board-certified surgeons and residents, the variance in their replies compared to ChatGPT-4 was more pronounced. There were significant overall tests (*p* < 0.001), with significant differences between board-certified surgeons and ChatGPT-4 (*p* < 0.003), residents, and ChatGPT-4 (*p* = 0.002), and board-certified surgeons and residents (*p* = 0.006), respectively.

C. In Case 3, the most valid answer was A, selected by board-certified surgeons. Residents chose two eligible answers, resulting in a significant difference between board-certified surgeons and residents (*p* = 0.002). In contrast, the significant differences between board-certified surgeons and ChatGPT-4 (*p* < 0.003) and between residents and ChatGPT-4 (*p* < 0.003) were less pronounced with significant overall tests (*p* < 0.001).

D. In Case 4, there was a high level of agreement between board-certified surgeons and residents, in contrast to ChatGPT-4. This resulted in significant differences between board-certified surgeons and ChatGPT-4 (*p* < 0.003) and between residents and ChatGPT-4 (*p* < 0.003), respectively. The overall tests were significant (*p* < 0.001).

Where significant results in the overall tests were followed up with post hoc pairwise group comparisons, Bonferroni adjustment was applied to *p* values to control for the increased risk of Type I errors due to multiple comparisons.

## 4. Discussion

The use of ChatGPT in plastic and reconstructive surgery is rapidly gaining interest as AI and Large Language Models (LLMs) demonstrate significant capabilities across various medical fields [3,10]. ChatGPT has shown potential in performing tasks ranging from generating literature reviews to aiding in clinical decision-making, and it is now being explored in surgical specialties such as plastic and reconstructive surgery.

LLMs demonstrate promise in administrative tasks, patient communication, and educational support. However, their limitations in medical accuracy and reliance on non-specialized datasets restrict their applicability for critical decision-making [3,10]. Task-specific LLMs trained on curated medical datasets could offer more reliable support, ensuring relevance and minimizing risks associated with erroneous outputs [3,10].

ChatGPT, as an LLM, has multiple applications in plastic surgery. It can assist surgeons in generating academic literature, enhancing productivity in scientific writing, and even helping with routine clinical documentation such as patient discharge summaries and operation notes. These functions can save significant time for clinicians, enabling them to focus on more complex tasks [5,11]. However, it is crucial to recognize the model’s limitation. Current iterations of ChatGPT are limited by their training data [5]. Additionally, while ChatGPT can generate content that sounds authoritative, it may produce inaccurate or fabricated information, raising concerns about its reliability in clinical settings [1,10].

Our study findings underline the limitations of AI tools specifically ChatGPT-4 in plastic surgery, particularly in handling complex reconstructions such as lower leg defects. The results reveal that ChatGPT-4’s responses to a complex problem differed significantly from the solutions proposed by board-certified plastic surgeons. Although ChatGPT-4 demonstrated proficiency in suggesting common solutions, it lacked the deep clinical understanding needed to address patient-specific variables like vascular occlusions or diabetes. This highlights the essential role of human expertise, especially when dealing with multifactorial patient scenarios, a point emphasized by Copeland-Halperin et al. (2023), who found that AI can support decision-making but struggles with nuanced clinical cases [8]. These findings align with Sharma et al. (2023), who highlighted the limited utility of AI in decision-heavy fields such as reconstructive surgery [5].

The observed limitations suggest that AI, while valuable in administrative tasks such as generating literature reviews or assisting in basic clinical documentation, must be used cautiously in clinical decision-making. For complex surgeries, the deep experiential knowledge of human surgeons remains irreplaceable. The integration of AI into surgical practice should be viewed as supplementary rather than substitutive. Recent studies by Leypold et al. (2023) and Liu et al. (2023) further emphasize the need for extensive validation and refinement of AI systems before they can reliably contribute to high-stakes decision-making [3,9]. Going forward, research should focus on creating more robust AI models capable of incorporating real-time clinical data and learning from case-based outcomes to match the decision-making capacity of experienced surgeons.

To optimize its outputs, careful prompt engineering is necessary, and responses need to be critically evaluated by clinicians before application [5]. This ensures that AI-generated suggestions are relevant and accurate for complex clinical decisions [3].

Copeland-Halperin et al. (2023) found that AI tools accurately retrieved information from authoritative sources such as the FDA and ASPS websites. However, they frequently failed to consistently provide correct answers for queries involving nuanced clinical decision-making [8].

Our results align with prior research and indicate that future progress in AI applications for plastic surgery will depend on developing algorithms that can selectively identify, assess, and filter information. Such advancements could enhance the accuracy, precision, validity, reliability, and clinical utility of AI-generated recommendations.

ChatGPT has potential in clinical decision support (CDS) by assisting surgeons with complex cases. For instance, it can generate suggestions to improve clinical decision logic in surgery, offering unique perspectives that complement human expertise [3]. In plastic surgery, this capability could prove particularly useful for intricate reconstructive procedures that require a deep understanding of patient-specific variables [9].

The significant differences observed between the recommendations of ChatGPT-4 and surgeons highlight the critical role of expert judgment in reconstructive surgery. For cases involving patients with complex comorbidities like vascular occlusions or diabetes, experienced surgeons were able to select the most appropriate techniques based on a comprehensive understanding of the patient’s condition and disease progression. This discrepancy underscores the limitations of current AI models, which lack the ability to integrate subtle clinical cues derived from years of hands-on experience. Constrained by their training data, AI systems often lack the contextual depth required for complex, multifactorial decision-making [8,9]. While AI can provide general suggestions, it falls short in the nuanced interpretation of individual cases. Recent studies echo these findings, demonstrating that while AI tools can enhance decision-making in routine, well-defined tasks, it struggles in areas requiring intuitive human judgment [5,8]. This reinforces the irreplaceable value of human expertise, particularly in complex surgical procedures, where decisions must be tailored to the individual needs of patients [3].

Beyond its limitation in direct clinical decision-making for complex reconstructive cases, ChatGPT offers significant potential in assisting with administrative and non-critical tasks. In fields such as plastic and reconstructive surgery, where documentation and communication consume a substantial amount of time, AI systems can automate routine tasks such as generating patient summaries, drafting operative reports, and even compiling literature reviews [3,10]. By taking on these responsibilities, AI has the potential to alleviate the workload on surgeons, allowing them to focus more on critical tasks that require specialized knowledge and skills [5,9]. It remains essential that these AI-generated documents are carefully reviewed and validated by human professionals, as current models may still produce errors or lack the precision required for complex medical documentation. Achieving a balance between AI support and human oversight will be crucial to maximizing the potential of AI tools in healthcare [1].

The integration of AI into clinical practice, especially in a nuanced field like plastic surgery, must be approached with caution. Ethical concerns regarding data privacy, the risk of misinformation, and the potential over-reliance on AI tools remain significant [2]. Surgeons must remain vigilant and ensure that AI tools like ChatGPT-4 are used as supplementary aids rather than replacements for human judgment. As AI technology evolves, its role in assisting with complex tasks in plastic and reconstructive surgery will likely expand, provided its limitations are addressed and that its outputs are closely monitored by experienced clinicians.

One limitation of this study was the small sample size and response rate. Nevertheless, we achieved sufficient statistical power and believe that these results are generalizable. A higher response rate could make the findings more representative. However, previous research suggests that physician surveys are less susceptible to non-response bias compared to surveys targeting other populations, as physicians typically exhibit relatively homogenous knowledge, attitudes, and beliefs [12].

Future studies should include a broader range of lower-limb defects to provide a more comprehensive representation of the diversity in lower-limb reconstruction scenarios. Moreover, it would be interesting to compare the capabilities of different AI tools, evaluate the significance of training, and explore the integration of various AI systems for different fields of expertise, such as those of board-certified medical specialists.

## 5. Conclusions

In this study, we aimed to and successfully demonstrated that ChatGPT-4 can evaluate various reconstruction methods and provide a response based on an image and case description. However, it lacks the expertise to draw the correct conclusions regarding the optimal reconstruction from the available information in both visual and written forms. Ultimately, plastic surgeons remain the gold standard for interpreting and managing the clinical picture in conjunction with the patient’s personal medical history. As of now, the application of AI in the field of decision-making surgery should focus on supporting the professionals rather than attempting to replace them.

## Figures and Tables

**Table 1:** Baseline demographic data.

Baseline Demographic Rater Data (*n* = 52)	
Characteristics	N ^1^ [Counts] (Percentage [%]), or Median (SD ^1^)
Gender	
Male	32 [63]
Female	18 [35]
Diverse	1 [2]
Status of training	
Board-certified surgeons	39 [75]
Residents	13 [25]
Board-certified surgeons (*n* = 39)	
Years after board certification	
<3 years	4 [10]
>3 years	35 [90]
Surgical spectrum	
Esthetic surgery	30 [77]
Reconstructive surgery	38 [97]
Microsurgery	32 [82]
Hand surgery	24 [62]
Burn surgery	14 [36]
Residents (*n* = 13)	
Years in training after common trunk	3 (1.58)
Surgical spectrum	
Esthetic surgery	9 [69]
Reconstructive surgery	13 [100]
Microsurgery	10 [77]
Hand surgery	9 [69]
Burn surgery	9 [69]

Continuous variables ± SD, binary variables, respectively, frequency tables. ^1^ N = total number of patients, SD = Standard deviation. Missing data for gender: 1.

**Table 2:** Answers Case 1.

Case 1	N ^1^ [Counts] (Percentage [%])
Answer A	
First step negative wound therapy; second step mesh graft	
Total	1
Board-certified surgeons	0 [0]
Residents	1 [10]
ChatGPT-4	0 [0]
Answer B	
Perforator propeller flap	
Total	6
Board-certified surgeons	2 [5]
Residents	4 [40]
ChatGPT-4	0 [0]
Answer C	
Free gracilis muscle flap	
Total	41
Board-certified surgeons	35 [95]
Residents	5 [50]
ChatGPT-4	1 [100]
Answer D	
Free temporoparietal fascia flap	
Total	0
Board-certified surgeons	0 [0]
Residents	0 [0]
ChatGPT-4	0 [0]

Binary variables; frequency tables. ^1^ N = total number of patients. Missing data for residents: 3; for board-certified surgeons: 2.

**Table 3:** Answers Case 2.

Case 2	N ^1^ [Counts] (Percentage [%])
Answer A	
First step negative wound therapy; second step mesh graft	
Total	7
Board-certified surgeons	7 [18]
Residents	0 [0]
ChatGPT-4	0 [0]
Answer B	
Pedicled instep (medial plantar artery) flap	
Total	1
Board-certified surgeons	0 [0]
Residents	0 [0]
ChatGPT-4	1 [100]
Answer C	
Superficial circumflex iliac artery (SCIP) flap	
Total	29
Board-certified surgeons	23 [61
Residents	6 [60]
ChatGPT-4	0 [0]
Answer D	
Free latissimus dorsi muscle flap	
Total	12
Board-certified surgeons	8 [21]
Residents	4 [40]
ChatGPT-4	0 [0]

Binary variables; frequency tables. ^1^ N = total number of patients. Missing data for residents: 3; for board-certified surgeons: 1.

**Table 4:** Answers Case 3.

Case 3	N ^1^ [Counts] (Percentage [%])
Answer A	
First step negative wound therapy; second step mesh graft	
Total	29
Board-certified surgeons	25 [66]
Residents	4 [40]
ChatGPT-4	0 [0]
Answer B	
Pedicled hemisoleus muscle flap	
Total	6
Board-certified surgeons	4 [11]
Residents	1 [10]
ChatGPT-4	1 [100]
Answer C	
Free split latissimus dorsi muscle flap	
Total	11
Board-certified surgeons	7 [18]
Residents	4 [40]
ChatGPT-4	0 [0]
Answer D	
Superficial circumflex iliac artery (SCIP) flap	
Total	3
Board-certified surgeons	2 [5]
Residents	1 [10]
ChatGPT-4	0 [0]

Binary variables; frequency tables. ^1^ N = total number of patients. Missing data for residents: 3; for board-certified surgeons: 1.

**Table 5:** Answers Case 4.

Case 4	N ^1^ [Counts] (Percentage [%])
Answer A	
Groin flap	
Total	1
Board-certified surgeons	1 [3]
Residents	0 [0]
ChatGPT-4	0 [0]
Answer B	
Free latissimus dorsi muscle flap	
Total	0
Board-certified surgeons	0 [0]
Residents	0 [0]
ChatGPT-4	0 [0]
Answer C	
Perforator propeller flap	
Total	2
Board-certified surgeons	0 [0]
Residents	1 [10]
ChatGPT-4	1 [100]
Answer D	
First step negative wound therapy, second step mesh graft	
Total	45
Board-certified surgeons	36 [97]
Residents	9 [90]
ChatGPT-4	0 [0]

Binary variables; frequency tables. ^1^ N = total number of patients. Missing data for residents: 3; for board-certified surgeons: 2.

## Data Availability

The original contributions presented in this study are included in the article. Further inquiries can be directed to the corresponding author.
